# Hysteresis of wettability in porous media: a review

**DOI:** 10.1007/s13202-020-00872-x

**Published:** 2020-04-02

**Authors:** Murtada A. Elhaj, M. Enamul Hossain, Syed A. Imtiaz, Greg F. Naterer

**Affiliations:** Memorial University of Newfoundland, St. John’s, Canada

**Keywords:** Contact angle, Hysteresis, Mathematical models, Experimental processes

## Abstract

The process of “hysteresis” has widely attracted the attention of researchers and investigators due to its usage in many disciplines of science and engineering. Economics, physics, chemistry, electrical, mechanical, and petroleum engineering are some examples of disciplines that encounter hysteresis. However, the meaning of hysteresis varies from one field to another, and therefore, many definitions occur for this phenomenon depending on the area of interest. The “hysteresis” phenomenon in petroleum engineering has gained the attention of researchers and investigators lately, because of the role that plays in reservoir engineering and reservoir simulation. Hysteretic effects influence reservoir performance. Therefore, an accurate estimation of rock and fluid property curves has an essential role in evaluating hydrocarbon recovery processes. In this paper, a comprehensive review of research and growth on the hysteresis of wettability for its applications in petroleum engineering is reported. Also, theoretical and experimental investigations of hysteresis of wettability are compared and discussed in detail. The review highlights a range of concepts in existing models and experimental processes for wettability hysteresis. Furthermore, this paper tracks the current development of hysteresis and provides insight for future trends in the research. Finally, it reveals an outlook on the research challenges and weaknesses of hysteresis of wettability.

## Introduction

Wettability of rocks is a crucial property in many aspects, such as controlling the location, flow, and distribution of fluids in the reservoir (Anderson 1986a). Moreover, studies have shown the effect of wettability in the electrical properties of porous media (Anderson 1986b; Elhaj et al. 2018a), capillary pressure (Anderson 1987a), waterflood behavior (Anderson 1987b), relative permeability (Anderson 1987c; Elhaj et al. 2018b), dispersion (Wang 1988), simulated tertiary recovery (Anderson 1986a), irreducible water saturation (Anderson 1987c), and residual oil saturation (Anderson 1987a; Hirasakl 1991). As it is known, wettability can be measured by contact angle (Yuan and Lee 2013; Zisman 1964), which has the ability to measure the angle of the wetting phase to solid. As the contact angle is a characteristic of the rock wettability, it is considered only an indication of rock wettability, which means a contact angle with much less than (90°) indicates high wettability. In contrast, the contact angle with a much larger angle than (90°) indicates low wettability. There are two types of contact angles: (1) static or (2) dynamic based basically on the movement or the stationary of the fluid and solid while the measurement takes place (Johnson et al. 1977). Most studies refer to wettability by the degree of contact angles (Michaels and Lummis 1959; Cassie and Baxter 1944; Bartell and Cardwell 1942). Based on this fact, the term “contact angle” used in this paper shall refer to wettability.

**Fig. 1:**
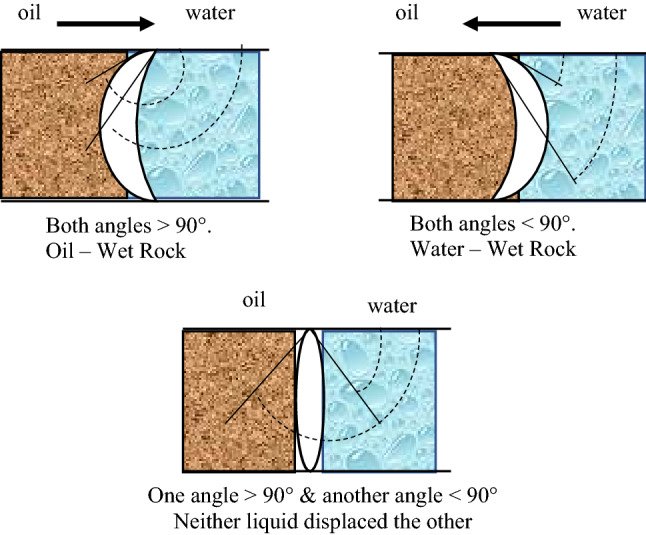
Schematic of three scenarios of hysteresis phenomenon in wettability (Benner et al. 1942)

The hysteresis of wettability has a long history in the oil and gas industry (Haines 1930; Benner et al. 1942; Melrose 1965). It was found in a previous study that the hysteresis that occurred in contact angle was akin to similar hysteresis that existed in petroleum engineering, such as capillary pressure hysteresis and relative permeability hysteresis (Johnson et al. 1977). Therefore, when the interface between oil and water, for instance, gave two angles versus reservoir rock, advancing and receding of the water, this phenomenon of exciting of two angles for one system is well known as hysteresis of contact angle (Benner et al. 1942). Other authors refer to the hysteresis term in wettability to the difference between these two angles (advancing and receding) (Gao and McCarthy 2006; Extrand 2003, 2002, 2004). Three cases can happen for a reservoir rock (Benner et al. 1942) which can be shown graphically in Fig. 1:

1. The two different angles were both less than 90°; the reservoir rock would be water-wet, and there would be a continuous movement of water forcing the oil out of the rock.
2. The two different angles were both higher than 90°; the reservoir rock would be oil-wet, and there would be a continuous movement of oil, forcing the water out of the rock.
3. The two different angles were on opposite sides (one was less than 90°, and the other was greater than 90°), and there would be no movement of liquid in either direction.

Despite the extensive studies that focused on investigating contact angle hysteresis, the fundamental reasons for this phenomenon are not entirely understood (Extrand and Kumagai 1997). It is often referred to as surface heterogeneity (Ruch and Bartell 1960; Good 1952; Pease 1945), roughness (Shuttleworth and Bailey 1948; Eick et al. 1975; Huh and Mason 1977), overturning of molecular segments at the surface (Langmuir 1938; Hansen and Miotto 1957; Ter-Minassian-Saraga 1964), adsorption and desorption (Vergelati et al. 1994), interdiffusion (Timmons and Zisman 1966; Good and Kotsidas 1979), and or surface deformation (Bikerman 1950; Lester 1961). In the next two sections, essential experimental and theoretical techniques will be highlighted and discussed.

## Physical explanation of hysteresis in wettability

To understand the physical cause of hysteresis in wettability, it is essential to have a good understanding of the physical explanation behind the occurrence of wettability itself. As it is known, the contact angle is considered one of the thermodynamic properties and it is commonly used to measure the wetting properties of two immiscible fluids (Xie et al. 2001). From a physical point of view, contact angle can be measured and defined using the term “surface energy” in Young’s equation (Xie et al. 2001; Ryder and Demond 2008). The contact angle is a function of three interfacial tension phases: (1) two-fluid phase, (2) solid drop phase, and (3) solid immersion phase.

Previous studies showed that contact angles measured macroscopically might differ from the intrinsic contact angle due to hysteresis phenomena (Eick et al. 1975; Ryder and Demond 2008; Dettre and Johnson 1965; Restagno et al. 2009). A justification of this phenomenon is that at a larger size of the drop, the advancing edge gives the contact angle against the low-energy areas of the surface. On the contrary, at a smaller size of the drop, the receding edge provides contact with the angle against the high-energy areas of the surface (Ryder and Demond 2008). Another physical justification of contact angle hysteresis occurrences is when a droplet experiences an external force which is considered extra energy of a system (Cheng et al. 2016). Moreover, molecular size and properties of liquid have also effect on contact angle hysteresis existence (Lam et al. 2001).

Several parameters and properties influence wettability hysteresis, as reported in many previous studies. These parameters are listed but not limited to surface roughness (Xie et al. 2001), surface geometries (Cheng et al. 2016), drop size (Brandon et al. 2003), liquid and solid surface composition (Ryder and Demond 2008), molecular size and properties of liquid (Lam et al. 2001), and solid–liquid contact time (Lam et al. 2002).

## Experimental observations of hysteresis in wettability

Many experimental techniques and methods were developed during the last decades to investigate and measure the hysteresis phenomenon in contact angles. These techniques can be divided into techniques that were measured on flat solid surfaces and others on different geometries (nonideal surfaces), such as plates, fibers, and powders (Chau 2009). In another perspective, these techniques can be categorized in terms of static and dynamic conditions depending on the situation of the liquids during measurements (Yuan and Lee 2013; Ralston and Newcombe 1992). In this section, both perspectives, movement type and surface type, will be discussed briefly.

The most common method that is used to describe and measure the contact angle depends on observing the image of the drop by low-magnification optical devices (Chau 2009). It is quite challenging to determine the degree of wettability with the low-magnification device. Additionally, keeping a surface clean in an open-air laboratory is almost an impossible task. An advantageous technique to keep surfaces clean and uncontaminated is abrasion and polishing underwater using scrupulously controlled conditions which is proposed and whose efficiency is proved (Wark and Cox 1932).

**Fig. 2:**
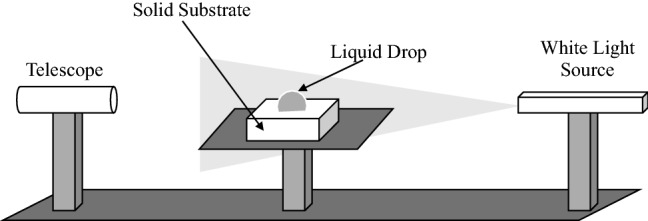
Sketch shows a telescope-goniometer technique for contact angle measurement (Salim et al. 2008)

A well-known technique that used to measure the tangent angle of the contact angle known as “telescope-goniometer” is used to determine the contact angles (Bigelow et al. 1946) on a flat solid surface, as shown in Fig. 2. The same method was designed and modified by Zisman (1968). The eyepiece was used to measure the tangent of the drop and the surface contact point. Over the years, enhancements and improvements were made to improve the accuracy of angle measurements, such as magnifying (up to 50 times) the intersection profile which allows for better assessment as well as using a camera instead (Smithwich 1988; Leja and Poling 1960). Another study proved the sessile drop’s angle could be measured up to the accuracy of ± 2° when the contact angle is higher than 20° (Hunter 2001). A motor-driven syringe is employed in the experimental setup to control the liquid rate when measuring the dynamic contact angle was another development for this technique (Kwok et al. 1996). The advantages of this method can be (1) simplicity, (2) small surface and a small amount of liquid are required to conduct this experiment. On the other hand, the disadvantages of this method can be summarized as follows:

1. As the liquid size and surface are small, the possibility of the existence of impurities which may affect the reading of the angle is likely to be high (Yuan and Lee 2013; Chau 2009).
2. This method entirely depends on the measurement of tangent line’s angle that leads to significantly inaccurate measurements if a minor error occurs (Yuan and Lee 2013).
3. The focus of the camera only is toward the most significant drops (Yuan and Lee 2013; Chau 2009).
4. Variations in contact angles’ measurements happen when the flat surface is either heterogeneous or rough (Chau 2009).
5. The small size of the droplet leads to difficulties in measuring the contact angle (Brandon et al. 2003; Letellier et al. 2007).

**Fig. 3:**
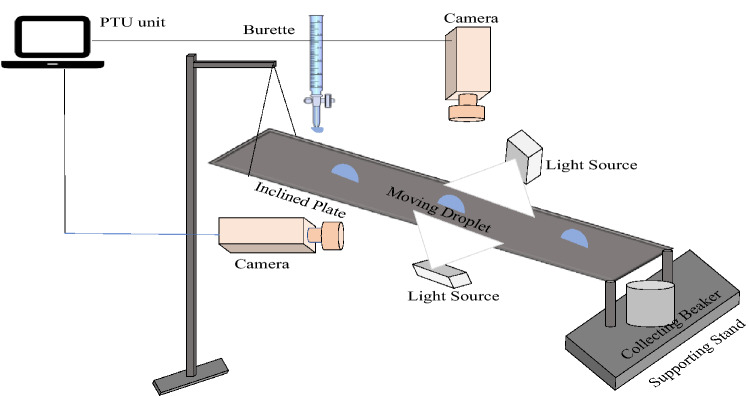
Sketch shows the inclined plate technique for contact angle measurement (Puthenveettil et al. 2013)

Another popular method that is used for investigating hysteresis is a “tilted plate” or “inclined plate” introduced in the 1940s (Macdougall and Ockrent 1942). Figure 3 depicts a schematic of the inclined plate method. This technique is a modified version of the “telescope-goniometer” technique. The same method was used to study contact angle hysteresis on various types of polymer surfaces, such as silicon wafers and elastometric surfaces (Extrand and Kumagai 1995, 1997). This technique used a recorded video camera and videotape to measure both angles using a protractor when the drop started moving; the tape was stopped. Measurements of these two angles must be taken carefully because most of the time, they can be different (Pierce et al. 2008; Krasovitski and Marmur 2005).

In the early history of contact angle measurement, a platinum wire was used to measure contact angle hysteresis by forming sessile drops on a solid surface (Zisman 1968). The drops were created by heating the wire and then putting it in a fluid to form the drops. The drop gently and slowly puts on a surface, building a sessile drop (Yuan and Lee 2013). Despite the accuracy of reproducing the sessile drop that was be claimed (± 2°) (Spelt et al. 1986), some concerns that moving the drop from the wire to the surface may cause some kinetic energy combined with the flowing, which may lead to metastable contact angles (Eick et al. 1975; Derjaguin 1946; Johnson and Dettre 1966; Neumann and Good 1972).

The tangentometer method is also known for measuring contact angle hysteresis, which uses a mirror that is seated at the baseline of the droplet (Yuan and Lee 2013; Phillips and Riddiford 1972). The role of the mirror is to rotate until the full curve of the drop is formed, and with its reflection image, the protractor that is adhered to the mirror can be used to measure the tangent line’s angle. This method has the problem of the measurement errors because of the inherent subjectivity of tangentometers (Fenrick 1964). The specular reflection from the drop surface by using a light source is another technique that can be applied to estimate the hysteresis of the contact angle (Langmuir and Schaefer 1937). The light source is rotated around the droplet until the reflection from the drop dies; afterward, the contact angle can be read from the degree of the rotation. The accuracy of this method is (± 1°) and can be used for both sessile drops and menisci (Johnson and Shah 1985).

Flat solid surfaces, horizontal or vertical, were the focus of the previous discussion, and general observations can be highlighted in these points:

1. Contact angle measurement relies mainly on two factors, which are the surface quality and its cleanliness (Chau 2009).
2. When the contact angle is under 20°, it is difficult to measure, and most of the techniques give inaccurate estimation (Gaudin 1957).
3. Heterogeneity of the surface appears to be the biggest problem for the flat surfaces’ measurement techniques (Extrand 2004; Neumann and Good 1972).
4. Some techniques use a small droplet and surface, which may lead to inaccuracy in measuring the contact angle hysteresis (Bigelow et al. 1946).

For the other type of surfaces, nonideal or different geometries, Table 1 summarizes, discusses, and analyzes the essential techniques that are used to measure contact angle hysteresis. In general comparison between these techniques, the most widely used technique that can be applied for most cases is the Wilhelmy balance method (Wilhelmy 1863) because it can be used in static and dynamic contact angle measurements and is simple. In addition, most of the other techniques primarily originated from its fundamentals. Other studies considered temperature dependency on measuring the contact angle hysteresis, such as the captive bubble method (Taggart et al. 1930) and capillary rise at a vertical plate method (Shimokawa and Takamura 1973; Neumann 1964; Budziak and Neumann 1990; Kwok et al. 1995). Some studies give very low error possibility, and others provide large error values under exceptional circumstances, such as capillary rise at a vertical plate method and individual fiber method (Schwartz and Minor 1959), respectively. For more details about these techniques that used experiments to estimate the contact angle, see Table 1.

**Table 1:** Review of nonideal geometry surfaces techniques for contact angle hysteresis

Technique name	Principles	Advantages	Disadvantages	References
Captive bubble technique	An air bubble is created below a solid surface by injecting air into a fluid A needle is used to keep the bubble from drifting	The surface is in contact with a saturated atmosphere Contaminated and clean surface Easy to study temperature dependence	It requires more liquid Liquid causes the sabotage of the film	Taggart et al. (1930)
Tilting plate method	A meniscus is created on both sides of the plate due to immersing a plate into a liquid The position of the meniscus to the plate must be horizontal The contact angle is the angle between the plate and the horizontal	It is simple and does not depend on the operator’s subjectivity Less error compared to others (only ± 5°)	The disturbance of the liquid during the measurement is considered to be a significant problem	Zhang et al. (1989)
Wilhelmy balance method	It moves flat plate up and down for measuring the contact angle It depends on surface tension calculations	Simplest Accurate contact angle values It is used for dynamic contact angle measurement	A high-sensitivity electrobalance is needed The plate must have a constant perimeter The sample must have the same composition and topography on all sides	Wilhelmy (1863), Santoso et al. (2019), Lund et al. 2019) and Rohmer et al. (2017)
Capillary rise at a vertical plate	Capillary height is determined by the integration of the Laplace equation Capillary height can be used to determine the contact angle.	Accuracy up to (± 0.1°) can be obtained The surface tension and contact angle can be measured at the same time The temperature versus contact angles can be measured	The surface tension should be known Inaccurate values if the liquid contains the surface-active agent	Shimokawa and Takamura (1973), Neumann (1964), Budziak and Neumann 1990) and Kwok et al. (1995)
Individual fiber	A fiber is put in a horizontal position in the microscopic field A goniometer eyepiece is a tool to estimate the contact angles	The zero contact angle can be measured The homogeneity of the fiber surface can be tested	Significant errors occurred due to the small dimensions of the drop curvature Practically, it is difficult to get accurate values when the depth of immersion is relatively small	Schwartz and Minor (1959)
Capillary penetration methods for particles	The rate of a liquid penetration is monitoring A flat cake is created by compressing the powders The contact angle is measured from the liquid drops	It gives better correlation results than the captive bubble technique It can handle the presence of a porous architecture	The method depends on determining the effective capillary radius, which is difficult to measure accurately The critical surface tension should always be higher than the surface tension The packing powder must be obtained in the capillary tubes	Washburn (1921), Zografi and Tam (1976) and Lerk et al. (1977)

## Modeling of hysteresis in wettability

As been discussed in previous sections, hysteresis can be referred to the difference between advancing $\theta_{\text{a}}$ and receding $\theta_{\text{r}}$ angles, which can be mathematically formulated as $\theta_{\text{hys}}$:

1 $$\theta_{\text{hys}} = \theta_{\text{a}} - \theta_{\text{r}}$$

The literature on contact angle hysteresis has highlighted several mathematical models. As Extrand (2003) reported, the first model was developed by Cassie and Baxter (1944) and Cassie (1948), which is applied to heterogeneous surfaces and can estimate the values of advancing and receding angles as:

2 $$\cos \theta_{i} = \alpha_{1} \cos \theta_{i,1} + \alpha_{2} \cos \theta_{i,2}$$

where *i* refers to either advancing or receding, for materials 1 and 2 , $\alpha_{1, }$ and $\alpha_{2}$ are the fractional areas of material 1 and material 2:

3 $$\alpha_{1} + \alpha_{2} = 1$$

The model developed by Cassie is simple and very straightforward, and the primary assumption of this model is that the fluid will change the model surfaces. Still, this model and other models that originated from it failed to predict contact angle correctly (Dettre and Johnson 1965; Gaines 1960; Brockway and Jones 1964) that is because all these models assumed that the apparent contact angle is controlled by the interfacial contact area between liquid and solid. Several studies have suggested that contact angles can be estimated by the interactions that occur at the contact line (Extrand 2002, 2003).

More advanced models have been developed by Good (1952), Neumann and Good (1972), Johnson and Dettre (1964), Öpik (2000) and Marmur (1994). Most of these models employed geometry as a function; moreover, the surface roughness was also included. The effect of surface roughness and chemical nonuniformities on the wettability hysteresis was investigated mathematically. In these mathematical models, the geometries were assumed to be regular, such as the form of parallel stripes (Öpik 2000). A previously published study that dealt with this assumption can be found in the Murmur's article, which contained a list of all previous references (Marmur 1994). The reader may also refer to the study done by de Genes for more details (De Gennes 1985).

An interesting study conducted by Brandon et al. (1997) modeled and simulated hysteresis phenomenon of three-dimensional sessile drops in equilibrium with a model of chemically heterogeneous smooth solid surface in which the energy is spatially periodic. The main assumptions of this model are: (1) the fluid and liquid are mutually immiscible, (2) gravity effect is neglected, and (3) contact angle is assumed to vary along the surface. To achieve stability, the dimensionless free energy of the system is given by:

4 $$G = S_{\text{IF}} - \left[ {{\iint }\cos \theta_{i} \left( {x,y} \right){\text{d}}x{\text{d}}y} \right]_{{S_{\text{SI}} }}$$

where *G* is free energy, *x* and *y* spatial coordinates, $S$ interfacial area, and $\cos \theta_{i} \left( {x,y} \right)$ can be defined by Young’s equation:

5 $$\cos \theta_{i} \left( {x,y} \right) = \frac{{\sigma_{\text{sf}} \left( {x,y} \right) - \sigma_{\text{sl}} \left( {x,y} \right)}}{{\sigma_{\text{lf}} }}$$

where $\sigma_{\text{sf}} \left( {x,y} \right) {\text{and}} \sigma_{\text{sl}} \left( {x,y} \right)$ are solid–liquid and solid–fluid interfacial tension, respectively. As a conclusion for this result, the hysteresis was found to have existed in both the average contact angle (as a function of volume) and liquid–fluid interfacial curvature. Another conclusion of this study was a good agreement in calculating the drop shapes in three-dimensional Young and Young–Laplace equations. Although this study gave good results as well as better understanding in three-dimensional point of view, it had limitations, that is, the software that was used failed to investigate a large drop size of a bubble, which is the same disadvantage of the study that dealt with two-dimensional sessile drop (Brandon and Marmur 1996). Several studies also considered the surface free energy of wetting as a function in mathematical models (Extrand 2002, 2003, 2004; Extrand and Kumagai 1997; Cheng et al. 2016; Extrand 1998).

## Summary and conclusions

Determination of solid surface tension is one of the most applications of wettability measurement, which was the focus of several studies for decades (Lam et al. 2002; Neumann and Good 1972; Marmur 1994; Brandon and Marmur 1996; Dettre and Johnson 1969). However, most existing techniques rely on surface deformation, not surface tension, except for indirect methods that can deal with surface tension (Kwok and Neumann 2003). The first model that correlated the contact angle and interfacial tension was proposed by Young. To test liquids on a solid surface, the surfaces need to be rigid, homogenous, smooth, and inert.

The main focus of most researchers when they studied the hysteresis of wettability was to allow a quick indication of surface hydrophobicity (Chau 2009). Numerous methods that are widely applied in measuring the contact angle hysteresis were discussed and analyzed, such as the conventional telescope-goniometer method, capillary penetration methods for particles, and the Wilhelmy balance method. The applications and setbacks of these techniques are shaded.

Each technique has its advantages and disadvantages, as can be seen in Table 1, but in general, the most widely used technique that can be applied for most cases is the Wilhelmy balance method (Wilhelmy 1863). On real mineral samples, researchers found that the accurate method to estimate the contact angle is capillary penetration because of its quickness and easiness compared to techniques on flat mineral surfaces (Chau 2009).

Numerous studies investigated and attempted to explain the reason for the existence of contact angle hysteresis mathematically and theoretically, which involved the drop volume (Marmur 1994), complex surface geometries (Cheng et al. 2016), and drop size (Brandon et al. 2003). However, the investigators concluded that the geometric characteristics of the patterned surface are one of the vital factors in measuring hysteresis of wettability. Despite all these studies, the hysteresis of the contact angle is still not fully understood.

Hysteresis is a natural phenomenon that occurs in many disciplines, such as economics, biology, chemistry, physics, mathematics, civil engineering, electrical engineering, and petroleum engineering. Each discipline has its definition, and applications of hysteresis depend on the nature of conditions (Elhaj et al. 2018a, b).

The focus of this paper has been on investigating the hysteresis phenomenon experimentally and theoretically in wettability. However, the discussion and investigation of this property revealed the gap in the part of either experiment, theoretical or mathematical, generally, can be highlighted as:

1. The limitations of the experimental studies such as special conditions, which made it inapplicable for others,
2. Most of the experiments are conducted in laboratory conditions, not reservoir conditions,
3. The mathematical models may have double integrals which makes it challenging to inverse the process mathematically, and
4. The analytical solution for such a model is complicated to be done, if not impossible.

## References

[CR1] Anderson WG (1986). Wettability literature survey-part 1: rock/oil/brine interactions and the effects of core handling on wettability. J Pet Technol.

[CR2] Anderson WG (1986). Wettability literature survey-part 3: the effects of wettability on the electrical properties of porous media. J Pet Technol.

[CR3] Anderson WG (1987). Wettability literature survey-part 4: effects of wettability on capillary pressure. J Pet Technol.

[CR4] Anderson WG (1987). Wettability literature survey-part 6: the effects of wettability on waterflooding. J Pet Technol.

[CR5] Anderson WG (1987). Wettability literature survey part 5: the effects of wettability on relative permeability. J Pet Technol.

[CR6] Bartell FE, Cardwell PH (1942). Reproducible contact angles on reproducible metal surfaces. I. Contact angles of water against silver and gold. J Am Chem Soc.

[CR7] Benner FC, Dodoo CG, Bartell FE (1942) Evaluation of effective displacement pressures for petroleum oil-water silica systems. In: Drilling and production practice, 1 January, New York, NY

[CR8] Bigelow WC, Pickett DL, Zisman WA (1946). Oleophobic monolayers: I. Films adsorbed from solution in non-polar liquids. J Colloid Sci.

[CR9] Bikerman JJ (1950). Sliding of drops from surfaces of different roughnesses. J Colloid Sci.

[CR10] Brandon S, Marmur A (1996). Simulation of contact angle hysteresis on chemically heterogeneous surfaces. J Colloid Interface Sci.

[CR11] Brandon S, Wachs A, Marmur A (1997). Simulated contact angle hysteresis of a three-dimensional drop on a chemically heterogeneous surface: a numerical example. J Colloid Interface Sci.

[CR12] Brandon S, Haimovich N, Yeger E, Marmur A (2003). Partial wetting of chemically patterned surfaces: the effect of drop size. J Colloid Interface Sci.

[CR13] Brockway LO, Jones RL (1964). Electron microscopic investigation of the adsorption of long-chain fatty acid monolayers on glass. Adv Chem.

[CR14] Budziak CJ, Neumann AW (1990). Automation of the capillary rise technique for measuring contact angles. Colloids Surf.

[CR15] Cassie ABD (1948). Contact angles. Discuss Faraday Soc.

[CR16] Cassie ABD, Baxter S (1944). Wettability of porous surfaces. Trans Faraday Soc.

[CR17] Chau TT (2009). A review of techniques for measurement of contact angles and their applicability on mineral surfaces. Miner Eng.

[CR18] Cheng BK, Naccarato B, Kim KJ, Kumar A (2016). Theoretical consideration of contact angle hysteresis using surface-energy-minimization methods. Int J Heat Mass Transf.

[CR19] De Gennes PG (1985). Wetting: statics and dynamics. Rev Mod Phys.

[CR20] Derjaguin BV (1946) On the dependence of the contact angle on the microrelief or roughness of a wetted solid surface. CR (Dokl) Acad Sci URSS 51

[CR21] Dettre RH, Johnson RE (1965). Contact angle hysteresis. IV. Contact angle measurements on heterogeneous surfaces. J Phys Chem.

[CR22] Dettre RH, Johnson RE (1969). Surface tensions of perfluoroalkanes and polytetrafluoroethylene. J Colloid Interface Sci.

[CR23] Eick JD, Good RJ, Neumann AW (1975). Thermodynamics of contact angles. II. Rough solid surfaces. J Colloid Interface Sci.

[CR24] Elhaj M, Barri A, Hashan M, Hossain ME (2018). State of the art on porosity and permeability hysteresis: useful techniques for hydrocarbon recovery. Soc Pet Eng.

[CR25] Elhaj M, Hashan M, Hossain ME (2018b) A critical review and future trend on relative permeability hysteresis. In: Society of petroleum engineers—SPE trinidad and tobago section energy resources conference 2018. 10.2118/191260-ms

[CR26] Extrand CW (1998). A thermodynamic model for contact angle hysteresis. J Colloid Interface Sci.

[CR27] Extrand CW (2002). Model for contact angles and hysteresis on rough and ultraphobic surfaces. Langmuir.

[CR28] Extrand CW (2003). Contact angles and hysteresis on surfaces with chemically heterogeneous islands. Langmuir.

[CR29] Extrand CW (2004). Contact angles and their hysteresis as a measure of liquid–solid adhesion. Langmuir.

[CR30] Extrand CW, Kumagai Y (1995). Liquid drops on an inclined plane: the relation between contact angles, drop shape, and retentive force. J Colloid Interface Sci.

[CR31] Extrand CW, Kumagai Y (1997). An experimental study of contact angle hysteresis. J Colloid Interface Sci.

[CR32] Fenrick WJ (1964). Simple tangentometer. Rev Sci Instrum.

[CR33] Gaines GL (1960). Some observations on monolayers of carbon-14 labeled stearic acid. J Colloid Sci.

[CR34] Gao L, McCarthy TJ (2006). Contact angle hysteresis explained. Langmuir.

[CR35] Gaudin AM (1957). Flotation.

[CR36] Good RJ (1952). A thermodynamic derivation of Wenzel’s modification of Young’s equation for contact angles; together with a theory of hysteresis^1^. J Am Chem Soc.

[CR37] Good RJ, Kotsidas ED (1979). Contact angles on swollen polymers: the surface energy of crosslinked polystyrene. J Adhes.

[CR38] Haines WB (1930). Studies in the physical properties of soil. V. The hysteresis effect in capillary properties, and the modes of moisture distribution associated therewith. J Agric Sci.

[CR39] Hansen RS, Miotto M (1957). Relaxation phenomena and contact angle hysteresis. J Am Chem Soc.

[CR40] Hirasakl GJ (1991). Wettability: fundamentals and surface forces. SPE J.

[CR41] Huh C, Mason SG (1977). Effects of surface roughness on wetting (theoretical). J Colloid Interface Sci.

[CR42] Hunter RJ (2001). Foundations of colloid science.

[CR43] Johnson RE, Dettre RH (1964). Contact angle hysteresis. III. Study of an idealized heterogeneous surface. J Phys Chem.

[CR44] Johnson RE, Dettre RH (1966). The wettability of low-energy liquid surfaces. J Colloid Interface Sci.

[CR45] Johnson KA, Shah DO (1985). Effect of oil chain length and electrolytes on water solubilization in alcohol-free pharmaceutical microemulsions. J Colloid Interface Sci.

[CR46] Johnson RE, Dettre RH, Brandreth DA (1977). Dynamic contact angles and contact angle hysteresis. J Colloid Interface Sci.

[CR47] Krasovitski B, Marmur A (2005). Drops down the hill: theoretical study of limiting contact angles and the hysteresis range on a tilted plate. Langmuir.

[CR48] Kwok DY, Neumann AW (2003). Contact angle, wettability and adhesion.

[CR49] Kwok DY, Budziak CJ, Neumann AW (1995). Measurements of static and low rate dynamic contact angles by means of an automated capillary rise technique. J Colloid Interface Sci.

[CR50] Kwok DY, Lin R, Mui M, Neumann AW (1996). Low-rate dynamic and static contact angles and the determination of solid surface tensions. Colloids Surf A Physicochem Eng Asp.

[CR51] Lam CN, Kim N, Hui D, Kwok DY, Hair ML, Neumann aW (2001). The effect of liquid properties to contact angle hysteresis. Colloids Surf A Physicochem Eng Asp.

[CR52] Lam CNC, Wu R, Li D, Hair ML, Neumann AW (2002). Study of the advancing and receding contact angles: liquid sorption as a cause of contact angle hysteresis. Adv Colloid Interface Sci.

[CR53] Langmuir I (1938). Overturning and anchoring of monolayers. Science (80-.).

[CR54] Langmuir I, Schaefer VJ (1937). The effect of dissolved salts on insoluble monolayers. J Am Chem Soc.

[CR55] Leja J, Poling GW (1960) On the interpretation of contact angle. In: Proceedings of the 5th mineral processing congress (IMM, London), pp 325

[CR56] Lerk CF, Lagas M, Boelstra JP, Broersma P (1977). Contact angles of pharmaceutical powders. J Pharm Sci.

[CR57] Lester GR (1961). Contact angles of liquids at deformable solid surfaces. J Colloid Sci.

[CR58] Letellier P, Mayaffre A, Turmine M (2007). Drop size effect on contact angle explained by nonextensive thermodynamics. Young’s equation revisited. J Colloid Interface Sci.

[CR59] Lund A, Dyke SJ, Song W, Bilionis I (2019). Global sensitivity analysis for the design of nonlinear identification experiments. Nonlinear Dyn.

[CR60] Macdougall G, Ockrent C (1942). Surface energy relations in liquid/solid systems. I. The adhesion of liquids to solids and a new method of determining the surface tension of liquids. Proc R Soc Lond A Math Phys Eng Sci.

[CR61] Marmur A (1994). Contact angle hysteresis on heterogeneous smooth surfaces. J Colloid Interface Sci.

[CR62] Melrose JC (1965). Wettability as related to capillary action in porous media. Soc Pet Eng J.

[CR63] Michaels AS, Lummis RC (1959) Contact angle hysteresis on aquagels. In: AIChE/SPE joint symposium on wetting and capillarity in fluid displacement processes, 17-20 May, Kansas City, Missouri, USA. 10.2118/1274-G

[CR64] Neumann AW (1964). Über die Meßmethodik zur Bestimmung grenzflächenenergetischer Größen. Zeitschrift für Phys Chemie.

[CR65] Neumann AW, Good RJ (1972). Thermodynamics of contact angles. I. Heterogeneous solid surfaces. J Colloid Interface Sci.

[CR66] Öpik U (2000). Contact-angle hysteresis caused by a random distribution of weak heterogeneities on a solid surface. J Colloid Interface Sci.

[CR67] Pease DC (1945). The significance of the contact angle in relation to the solid surface. J Phys Chem.

[CR68] Phillips MC, Riddiford AC (1972). Dynamic contact angles. II. Velocity and relaxation effects for various liquids. J Colloid Interface Sci.

[CR69] Pierce E, Carmona FJ, Amirfazli A (2008). Understanding of sliding and contact angle results in tilted plate experiments. Colloids Surf A Physicochem Eng Asp.

[CR70] Puthenveettil BA, Senthilkumar VK, Hopfinger EJ (2013). Motion of drops on inclined surfaces in the inertial regime. J Fluid Mech.

[CR71] Ralston J, Newcombe G (1992) Static and dynamic contact angles. In: Laskowski JS, Ralston J (eds) Colloid chemistry in mineral processing, Elsevier, p 173. https://www.sciencedirect.com/science/article/pii/B9780444882844500020

[CR72] Restagno F, Poulard C, Cohen C, Vagharchakian L, Léger L (2009). Contact angle and contact angle hysteresis measurements using the capillary bridge technique. Langmuir.

[CR73] Rohmer J, Loschetter A, Raucoules D (2017) Global sensitivity analysis for supporting history matching of geomechanical reservoir models using satellite InSAR data: a case study at the CO_2_ storage site of In Salah, Algeria. In: Sensitivity analysis in earth observation modelling, pp 145–159 10.1016/B978-0-12-803011-0.00008-2

[CR74] Ruch RJ, Bartell LS (1960). Wetting of solids by solutions as a function of solute adsorption1, 2. J Phys Chem.

[CR75] Ryder JL, Demond AH (2008). Wettability hysteresis and its implications for DNAPL source zone distribution. J Contam Hydrol.

[CR76] Salim A, Sausse J, Pironon J, Fourar M, de Veslud CLC (2008). 3D confocal scanning laser microscopy to quantify contact angles in natural oil–water mixtures. Oil Gas Sci Technol.

[CR77] Santoso R, Hoteit H, Vahrenkamp V (2019) Optimization of energy recovery from geothermal reservoirs undergoing re-injection: conceptual application in Saudi Arabia. In: SPE middle east oil and gas show and conference. 10.2118/195155-MS

[CR78] Schwartz AM, Minor FW (1959). A simplified thermodynamic approach to capillarity: I. Application to flow in capillary channels. J Colloid Sci.

[CR79] Shimokawa M, Takamura T (1973). Relation between interfacial tension and capillary liquid rise on polished metal electrodes. J Electroanal Chem Interfacial Electrochem.

[CR80] Shuttleworth R, Bailey GLJ (1948). The spreading of a liquid over a rough solid. Discuss Faraday Soc.

[CR81] Smithwich RW (1988). Contact-angle studies of microscopic mercury droplets on glass. J Colloid Interface Sci.

[CR82] Spelt JK, Absolom DR, Neumann AW (1986). Solid surface tension: the interpretation of contact angles by the equation of state approach and the theory of surface tension components. Langmuir.

[CR83] Taggart AF, Taylor TC, Ince CR (1930). Experiments with flotation agents. Trans Am Inst Min Metall Eng.

[CR84] Ter-Minassian-Saraga LISBETH (1964). Chemisorption and dewetting of glass and silica. Adv Chem.

[CR85] Timmons CO, Zisman WA (1966). The effect of liquid structure on contact angle hysteresis. J Colloid Interface Sci.

[CR86] Vergelati C, Perwuelz A, Vovelle L, Romero MA, Holl Y (1994). Poly (ethylene terephthalate) surface dynamics in air and water studied by tensiometry and molecular modelling. Polymer (Guildf).

[CR87] Wang FHL (1988). Effect of wettability alteration on water-oil relative permeability, dispersion, and flowable saturation in porous media. SPE Reserv Eng.

[CR88] Wark SI, Cox AB (1932) Principles of flotation: an experimental study of the effect of xanthates on contact angles at mineral surfaces. American Institute of Mining & Metallurgical Engineers

[CR89] Washburn EW (1921). The dynamics of capillary flow. Phys Rev.

[CR90] Wilhelmy L (1863). Ueber die Abhängigkeit der Capillaritäts-Constanten des Alkohols von Substanz und Gestalt des benetzten festen Körpers. Ann Phys.

[CR91] Xie X, Morrow NR, Buckley JS (2001). Contact angle hysteresis and the stability of wetting changes induced by adsorption from crude oil. J Pet Sci Eng.

[CR92] Yuan Y, Lee TR, Bracco G, Holst B (2013). Contact angle and wetting properties. Surface science techniques.

[CR93] Zhang W, Wahlgren M, Sivik B (1989). Membrane characterization by the contact angle technique: II. Characterization of UF-membranes and comparison between the captive bubble and sessile drop as methods to obtain water contact angles. Desalination.

[CR94] Zisman WA (1964). Relation of the equilibrium contact angle to liquid and solid constitution. Am Chem Soc Publ.

[CR95] Zisman WA (1968). The solid/liquid interface—an essential and active frontier of science. Adv Chem.

[CR96] Zografi G, Tam SS (1976). Wettability of pharmaceutical solids: estimates of solid surface polarity. J Pharm Sci.

